# Uncovering mental well-being profiles in urban slums of Gorakhpur, India: A cluster-based approach using SWEMWBS

**DOI:** 10.1017/gmh.2026.10132

**Published:** 2026-01-26

**Authors:** U. Venkatesh, Arshad Ahmed, Ashoo Grover, Ashish Joshi, Om Prakash Bera, Anand Mohan Dixit, Hari Shanker Joshi, R. Durga

**Affiliations:** 1 Department of Community & Family Medicine, All India Institute of Medical Sciences, Gorakhpur, India; 2 Indian Council of Medical Research, New Delhi, India; 3 The University of Memphis School of Public Health, USA; 4 Global Health Advocacy Incubator, (GHAI), Washington, DC, USA; 5 Indian Council of Medical Research, Regional Medical Research Centre, Gorakhpur, India; 6 Independent Researcher, Gorakhpur, India

**Keywords:** mental well-being, SWEMWBS, urban slums, cluster analysis, India, psychological distress, public mental health.

## Abstract

Mental well-being is a growing but underrecognized public health priority in rapidly urbanizing, resource-constrained settings. Conventional mean-based analyses obscure important heterogeneity within vulnerable populations. We aimed to identify distinct mental well-being profiles among adults living in urban slums of Gorakhpur, India, using a person-centered approach. A cross-sectional survey (2023–2024) was conducted among 406 adults (≥18 years) from eight randomly selected slum settlements. Mental well-being was measured using the Short Warwick–Edinburgh Mental Well-being Scale (SWEMWBS). Standardized item scores were analyzed using K-means clustering, with the optimal cluster solution determined by the elbow method and validated using silhouette and Davies–Bouldin indices. Associations with sociodemographic and psychological factors were examined using chi-square tests, ANOVA, and multiple linear regression. Three profiles emerged: High (n = 133), Moderate (n = 137), and Low well-being (n = 136). SWEMWBS scores differed significantly across clusters (F(2,403) = 482.1; p < 0.001). The Low well-being group reported substantially higher stress, depression, and anxiety, and women were disproportionately represented (*χ* ^2^(2) = 29.30; p < 0.001). Longer sleep duration, higher household education, and lower stress independently predicted better wellbeing. Mental well-being is highly heterogeneous within urban slum populations. Cluster-based profiling enables more precise, equitable, and context-sensitive mental health interventions.

## Graphical abstract

**Figure:**
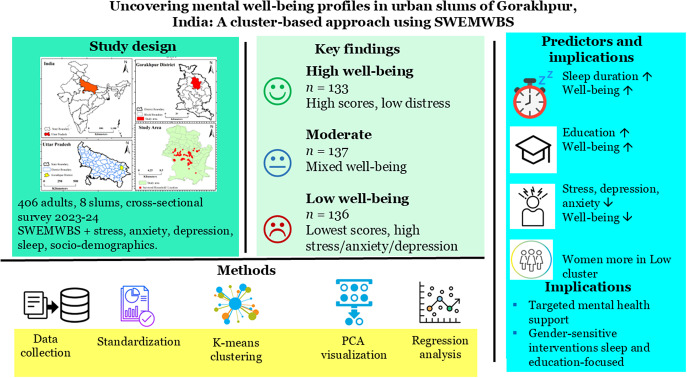

## Impact statements

Mental well-being is a growing public health concern in rapidly urbanizing Indian cities, particularly in slum communities where daily stressors, poverty and limited access to services may undermine psychological health. Yet, most existing research reports only average mental well-being scores, masking important differences between individuals. This study provides a cluster-based analysis of mental well-being among adults living in urban slums of Gorakhpur, uncovering three distinct groups: High, Moderate and Low well-being. This person-centered approach has several important implications. First, it shows that psychological health varies substantially even within socioeconomically similar populations. Identifying subgroups helps public health programs move beyond “one-size-fits-all” models and design targeted interventions that better match community needs. For example, those in the Low well-being cluster may benefit from focused mental health screening, stress-management support and gender-sensitive outreach, especially because women were more likely to fall in this group. The Moderate cluster, often overlooked, may profit from resilience-building programs, while those with high well-being could serve as peer supporters within community networks. Second, the strong link observed between sleep duration, education and mental well-being highlights modifiable behavioral and social levers that can be incorporated into routine public health practice. Sleep hygiene promotion, adult education and awareness initiatives may have meaningful psychological benefits when integrated into existing primary health systems. Third, the study supports the growing relevance of digital literacy in mental health. As health services increasingly adopt digital platforms, ensuring digital inclusion may improve access to information, self-care tools and support services for vulnerable populations. Overall, this research demonstrates the value of using cluster-based profiling to understand diverse mental health needs in underserved communities and provides actionable insights for designing equitable, scalable and context-sensitive mental health programs in low-resource urban settings.

## Introduction

Mental well-being is increasingly recognized as a key determinant of public health, encompassing not just the absence of mental illness but also the presence of positive psychological functioning (Barry, 2009; Slade, 2010). The World Health Organization defines mental well-being as a state wherein individuals realize their abilities, cope with normal life stresses, work productively and contribute to their communities (WHO, 2022). This broad understanding integrates both hedonic well-being (pleasure and happiness) and eudaimonic well-being (purpose and self-realization), aligning with the dual continua model, which emphasizes that well-being and mental illness are correlated but separate constructs (Gautam et al., 2024; Joshanloo, 2024).

In low- and middle-income countries (LMICs) such as India, there is growing recognition of the mental health burden, especially in urban populations living in informal settlements or slums. These communities are exposed to a range of psychosocial stressors, including overcrowding, poor sanitation, financial instability and limited access to healthcare, which can erode psychological resilience (Ezeh et al., 2017; Abdulhadi et al., 2024; Koly et al., 2025). Despite this, the literature on mental health in such populations remains disproportionately focused on clinical disorders like depression and anxiety, often neglecting the broader spectrum of positive mental well-being (Kirkbride et al., 2024).

Further complicating the picture is the widespread reliance on symptom-based or deficit-oriented tools for assessment, which may underrepresent individuals experiencing subclinical but meaningful psychological distress (Newson et al., 2020; MacNeill et al., 2021). In response, population-level tools, such as the Short Warwick-Edinburgh Mental Well-being Scale (SWEMWBS), have been developed to assess mental functioning on a positive spectrum (Shah et al., 2021; Pakpour et al., 2024). The SWEMWBS captures essential facets, such as optimism, perceived usefulness, clarity of thought and emotional connection, providing a holistic snapshot of mental wellness (Tennant et al., 2007).

However, most studies employing SWEMWBS or similar instruments report average scores or simple correlational analyses, thereby overlooking the underlying heterogeneity in patterns of mental well-being (Yang et al., 2024). In diverse and vulnerable populations, such as urban slum dwellers, mean-based analyses may mask latent subgroups with unique psychosocial profiles. This limitation calls for more nuanced, person-centered methodologies. The dual-continua model provides an important theoretical basis for employing a person-centered analytic approach. Because the model conceptualizes mental well-being and mental distress as related but distinct dimensions, it implies that individuals may cluster into qualitatively different well-being profiles rather than forming a single continuum. This framework, therefore, supports the use of cluster analysis to empirically identify subgroups that simultaneously reflect strengths and vulnerabilities. In line with this model, we expected that distinct latent profiles would emerge, capturing variations in emotional, cognitive and functional aspects of well-being among slum-dwelling adults.

Cluster analysis offers a compelling solution. Unlike regression models that assume uniform effects across a population, clustering techniques identify empirically derived subgroups based on shared internal characteristics (Gao et al., 2023). These subgroups, or “mental well-being profiles,” can capture co-occurring dimensions of strength and vulnerability, allowing for a more targeted and equitable approach to mental health promotion. International studies have demonstrated the value of such methods (Blumberg and Luke, 2009; Ueno et al., 2025). For instance, the UPRIGHT Project identified adolescent mental health subtypes in five European countries based on well-being, resilience and symptoms of distress, showing how school-based interventions could be tailored by profile (on behalf of the UPRIGHT consortium et al., 2019). Similarly, the HABITAT longitudinal study in Australia applied SWEMWBS to examine changes in mental well-being among older adults in relation to environmental exposures like greenspace (Carver et al., 2024).

Yet, such approaches remain rare in LMICs. In particular, urban slum populations in India are largely absent from research that integrates positive mental health measurement with multivariate statistical modeling (Maitra et al., 2015; Nolan, 2015). This gap is significant not only because these communities are socioeconomically marginalized but also because they are rarely the focus of data-driven mental health segmentation that could inform tailored, resource-efficient interventions.

Another understudied area is the intersection between technology use and mental well-being. With the rapid digitization of public health, understanding how digital literacy and mobile device proficiency relate to psychological wellness in low-income populations is both timely and policy-relevant (Ahmed et al., 2023). While some studies suggest that digital access can facilitate social connectedness and self-efficacy, others point to the risks of digital exclusion, especially among women and older adults (Sun et al., 2022; Venkatesh et al., 2022; Arias López et al., 2023).

Given these contextual, methodological and thematic gaps, there is a compelling need for studies that move beyond mean comparisons and explore typologies of mental well-being using clustering techniques in vulnerable urban settings. Doing so not only advances theoretical understanding of mental health heterogeneity but also holds practical value for public health systems aiming to implement tiered or targeted mental health interventions in under-resourced environments.

While several studies in India have examined mental health challenges in slum settings, very limited work has explored positive mental well-being through a person-centered, cluster-analytic lens. This methodological gap restricts the ability of public health systems to identify meaningful subgroups and design targeted, data-driven mental health interventions. Therefore, the present study focuses on uncovering underlying heterogeneity in mental well-being among adults living in urban slums of Gorakhpur.

Accordingly, this study had two primary aims: (1) to identify latent mental well-being profiles using item-level clustering of the SWEMWBS, and (2) to examine how these profiles differ across sociodemographic, behavioral and psychological factors. Based on prior literature, we hypothesized that: (a) multiple distinct well-being clusters would emerge rather than a single uniform pattern; (b) lower education, shorter sleep duration and higher psychological distress would be associated with poorer well-being clusters; and (c) women would be more likely to belong to lower well-being profiles.

## Methodology

### Study design and setting

This cross-sectional quantitative study was conducted between 2023 and 2024 to validate the SWEMWBS among adults residing in urban slums of Gorakhpur, Uttar Pradesh, India. The study is nested within a larger mixed-methods research project focused on physical, mental and social well-being in underprivileged urban populations.

### Study area

**Figure 1.**
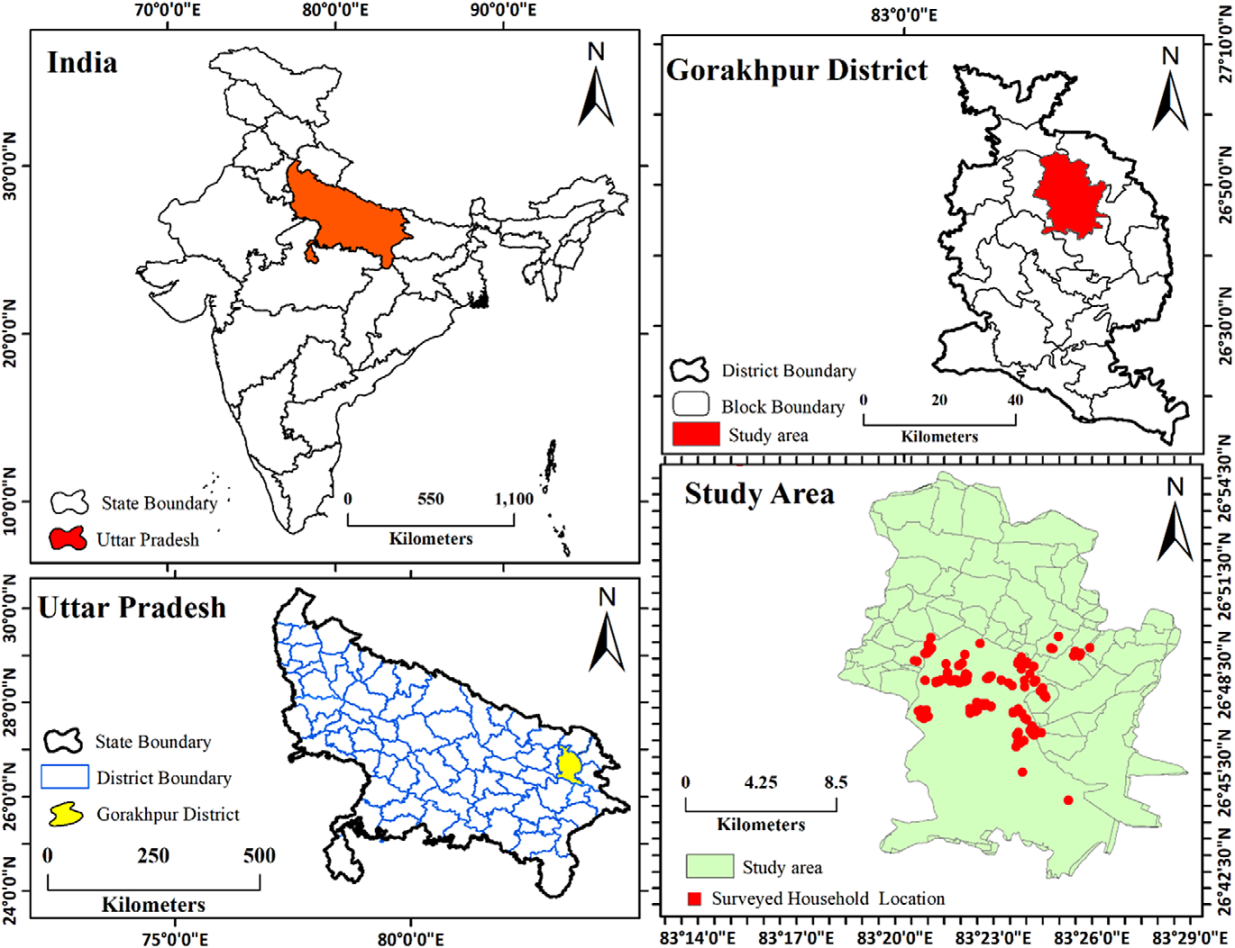
Location of the study area.

Gorakhpur Urban Agglomeration encompasses the municipal corporation area and surrounding Air Force zones, covering ~142.13 km^2^ with an estimated population of 671,048 (Figure 1). According to the 2011 Census and subsequent field surveys, ~67% of the urban population lives in slum and semi-slum settlements characterized by overcrowding, limited access to basic amenities and socioeconomic deprivation. The sex ratio stands at 944 females per 1,000 males, with an average literacy rate of 82.11%. Gorakhpur’s urban population growth rate has been driven by both natural increase and migration from surrounding districts, such as Deoria, Basti, Ballia and others. Notably, many slum settlements remain unregistered, limiting access to public services. The selected slums represented typical informal settlements commonly found in medium-sized cities of Uttar Pradesh. These included predominantly non-notified and unauthorized slum clusters lacking formal land tenure, consistent access to basic services and planned resettlement infrastructure. Unlike large metropolitan areas where categories such as JJ clusters or resettlement colonies are well-documented, Gorakhpur’s slum landscape is characterized mainly by organically formed, high-density settlements with limited municipal recognition. All eight selected slums shared broadly similar socioeconomic and environmental conditions, including overcrowding, poor sanitation, irregular housing structures and dependence on informal labor, thereby providing a relatively homogeneous context for analyzing mental well-being patterns.

### Participants

The present study was conducted among adults residing in urban poor settings of Gorakhpur. The study population included individuals aged 18 years and above, living in these underprivileged urban areas. Participants were enrolled based on specific inclusion and exclusion criteria. The inclusion criteria were as follows: (a) adults aged 18 years and above, (b) individuals who had given informed consent to participate in the study, (c) those available for a follow-up interview, (d) individuals experiencing physical, mental and social well-being issues and (e) those possessing an Android phone and willing to use technology for the purpose of the study.

Exclusion criteria included: (a) individuals below 18 years of age, (b) persons with terminal illness, (c) individuals with impaired cognition, (d) pregnant or lactating women, (e) those currently involved in other clinical trials or protocols related to well-being and (f) individuals with other limitations judged to interfere with study participation or the ability to follow study procedures, such as scheduled surgery, travel plans or scheduling difficulties that do not permit full participation. We acknowledge that requiring participants to own an Android phone may have systematically excluded individuals with the lowest socioeconomic status, including those lacking digital access, which has implications for representativeness.

### Sampling and data collection

A multistage random sampling technique was used to select eight urban slums across the four city zones (North, South, East and West). Households were randomly selected within each slum, and one eligible adult was enrolled per household. Within each selected slum, households were approached using a systematic walk-through approach starting from a central landmark. Every *n*th household was invited to participate, and if no eligible adult was available, the next household was approached. This procedure ensured that household selection remained random and evenly distributed across the slum area. The initial target sample size of 480 participants (30 per slum over two planned survey rounds: 30 × 8 × 2 = 480) was determined based on feasibility recommendations for pilot and psychometric studies in low-resource urban settings (Billingham et al., 2013). The “×2” reflected two planned data-collection rounds under the study framework. For assessing statistical adequacy, we followed established recommendations for psychometric and multivariable analyses. Guidelines for scale validation typically suggest an item-to-participant ratio of at least 1:30, with 200–300 participants considered adequate for stable factor structures and reliability estimates (MacCallum et al., 1999; Comrey and Lee, 2013). With seven SWEMWBS items, our final sample of 406 participants exceeded these thresholds and, therefore, provided sufficient power for both regression and *K*-means clustering. Prior methodological work also indicates that cluster solutions become stable and reproducible once sample sizes are above 300, especially when clustering is based on continuous variables. We did not pursue a larger sample due to practical challenges of conducting household-based surveys in dense, highly mobile slum environments, including limited daytime availability of eligible adults and operational constraints of the wider project. At the same time, we avoided a smaller sample to prevent reduced statistical precision, unstable cluster solutions and loss of representativeness. Although the achieved sample decreased from 480 to 406 due to nonresponse and data cleaning, proportional sampling across all four municipal zones was maintained. This ensured that the final sample continued to reflect the spatial distribution and demographic diversity of Gorakhpur’s slum population. Data were collected through face-to-face interviews conducted by trained fieldworkers to assist with literacy challenges.

### Measurement tool

The SWEMWBS is a validated instrument comprising seven positively worded items reflecting aspects of positive mental functioning. Items include: (i) I’ve been feeling optimistic about the future; (ii) I’ve been feeling useful; (iii) I’ve been feeling relaxed; (iv) I’ve been dealing with problems well; (v) I’ve been thinking clearly; (vi) I’ve been feeling close to other people; and (vii) I’ve been able to make up my own mind about things. Each item is rated on a 5-point Likert scale (1 = *None of the time* to 5 = *All of the time*), yielding a total score ranging from 7 to 35, with higher scores indicating better mental well-being (Tennant et al., 2007). The internal consistency of the SWEMWBS in this sample was excellent (Cronbach’s *α* = 0.922).

### Descriptive and inferential statistics

Descriptive statistics (mean, standard deviation [SD], skewness, kurtosis, histograms and *Q*–*Q* plots) were computed to assess the distribution of SWEMWBS scores. The distribution showed non-normality, evident from visual inspection and bimodal tendencies, prompting the use of nonparametric tests for group comparisons.

### Bivariate analysis

*T*-tests and analysis of variance (ANOVA) were used to compare SWEMWBS scores across gender and education levels. Chi-square tests assessed associations between categorical variables such as alcohol use and sleep quality/duration.

### Cluster analysis of mental well-being profiles

To explore heterogeneity in mental well-being, a *K*-means cluster analysis was conducted using the seven standardized (*z*-scores) SWEMWBS items as input features.

1. ***Standardization:***

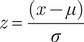

where 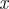 = individual item score, 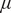 = mean and 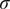 = standard deviation.

1. ***Determining optimal clusters:***

The elbow method was used by plotting the within-cluster sum of squares against increasing values of 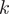 , with a clear inflection observed at 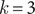 . To further validate the robustness of this choice, we computed two additional cluster validation indices: the average silhouette coefficient and the Davies–Bouldin index. Higher silhouette values and lower Davies–Bouldin scores indicated that *k* = 3 provided the best separation and compactness compared with alternative cluster solutions (*k* = 2–6).

1. ***Clustering execution:***

*K*-means was applied with: 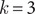 , 25 random initializations to avoid local minima, Euclidean distance as the similarity metric.

1. ***Visualization*:**

A principal component analysis was performed to reduce dimensionality for two-dimensional (2D) visualization, showing clear separation among the three clusters.

### Cluster profiles

Cluster 1 (Low well-being): Low scores on all SWEMWBS items, high levels of stress, depression and anxiety. Cluster 2 (Moderate well-being): Mixed scores and moderate distress levels. Cluster 3 (High well-being): High scores across all items and minimal psychological distress.

### Regression analysis

To identify the predictors of mental well-being, a multiple linear regression model was developed using the total score on the SWEMWBS as the dependent variable. The SWEMWBS is a validated continuous measure of positive mental health. A total of 10 independent variables were included in the model, selected based on their theoretical importance and empirical support from prior literature (Tennant et al., 2007; Shah et al., 2021; Pakpour et al., 2024). These predictors were gender, age, monthly income, education level of the head of household, smoking status, alcohol use, self-reported sleep duration, stress score, depression score and anxiety score.

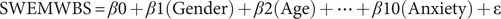



### Software

Statistical analyses were performed in R version 4.5.0 (R Foundation for Statistical Computing, Vienna, Austria). Regression models, ANOVA, chi-square tests, correlation analysis and descriptive statistics were conducted using the stats package, while *K*-means clustering and visualization of cluster structure were implemented using the cluster and factoextra packages. The study area map was prepared using QGIS (version 3.x).

### Variable coding

Gender was coded as a binary variable (0 = Female, 1 = Male), and age was treated as a continuous variable in years. Monthly income was also treated as a continuous measure, expressed in Indian Rupees. Education level was coded ordinally on a scale from 1 to 7: 1 for illiterate, 2 for primary school, 3 for middle school, 4 for high school, 5 for intermediate/diploma, 6 for graduate and 7 for professional degree. Both smoking status and alcohol use were coded as binary variables (1 = Yes, 2 = No). Sleep behavior was assessed using two variables: a binary indicator of whether the individual reports sleeping well (1 = Yes, 2 = No), and sleep duration categorized as an ordinal variable (1 = <5 h, 2 = 5 to <7 h, 3 = 7 or more hours). Stress, depression and anxiety scores were treated as continuous variables derived from validated psychometric instruments used in the broader study protocol.

All independent variables were entered into the regression model simultaneously using the Enter method (standard multiple regression), which allows for the assessment of each predictor’s unique contribution while controlling for others. Before analysis, standard assumptions of linear regression were evaluated. Linearity between continuous predictors and the dependent variable was examined through scatterplots. Multicollinearity was assessed using the variance inflation factor, with a cutoff value of 5; no predictor exceeded this threshold. Homoscedasticity of residuals was evaluated via residual plots and confirmed by the Breusch–Pagan test. The normality of residuals was assessed using *Q*–*Q* plots and the Shapiro–Wilk test, and independence of errors was tested using the Durbin–Watson statistic. All statistical analyses were performed using R software (version 4.3.1), with a two-tailed significance level set at *p* < .05.

### Ethical considerations

The study received ethical clearance from the Institutional Human Ethics Committee of AIIMS, Gorakhpur (Ref No: IHEC/AIIMS-GKP/BMR/109/2022). Written informed consent was obtained from all participants. For illiterate individuals, the consent form was read aloud in the presence of a witness, and consent was recorded.

## Results

### Participant characteristics

A total of 406 adults residing in urban slum settlements of Gorakhpur, Uttar Pradesh, were included in the analysis. As shown in Table 1, the sample was almost evenly distributed by gender, with 52.5% males and 47.5% females. Most participants were currently married and cohabiting (70.9%), with a minority never married (26.1%). The majority belonged to joint families (82%) and were classified as belonging to the “Upper Lower” (70.2%) or “Lower” (21.7%) socioeconomic strata, based on the modified Kuppuswamy scale. A large proportion identified as Hindu (94.1%), with Muslims comprising the remainder.

**Table 1.** Sociodemographic characteristics of the study participants (*N* = 406)

Characteristic	Category	Frequency (*n*)	Percentage (%)
Gender	Male	213	52.5
	Female	193	47.5
Marital status	Never married	106	26.1
	Married and cohabiting	288	70.9
	Married but not Cohabiting	10	2.5
	Divorced/widowed	2	0.5
Type of family	Joint	333	82.0
	Nuclear	73	18.0
Socioeconomic status (Kuppuswamy scale)	Upper	0	0.0
	Upper middle	5	1.2
	Lower middle	28	6.9
	Upper lower	285	70.2
	Lower	88	21.7
Religion	Hindu	382	94.1
	Muslim	24	5.9
	Christian	0	0.0
	Others	0	0.0

### Scale reliability and distribution

The SWEMWBS demonstrated excellent internal consistency in the study population (Cronbach’s *α* = 0.922). Corrected item-total correlations ranged from 0.659 to 0.804, confirming that all items contributed meaningfully to the scale (Table 2).

**Table 2.** Internal consistency of the SWEMWBS (Cronbach’s *α*)

Item	Mean	SD	Corrected item-Total correlation	Cronbach’s *α* if item deleted
Feeling optimistic about the future	3.63	1.23	0.762	0.909
I’ve been feeling useful	3.61	1.23	0.766	0.908
I’ve been feeling relaxed	3.53	1.31	0.760	0.909
I’ve been dealing with problems well	3.57	1.25	0.794	0.906
I’ve been thinking clearly	3.49	1.30	0.804	0.904
I’ve been feeling close to other people	3.31	1.15	0.742	0.911
I’ve been able to make up my own mind about things	3.25	1.15	0.659	0.919
Overall scale	24.39	7.11	-	*α* = 0.922

**Figure 2.**
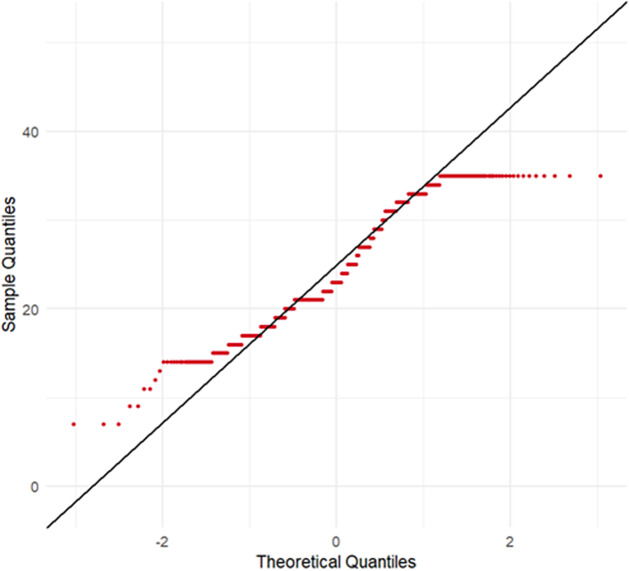
*Q*–*Q* plot of SWEMWBS scores.

**Figure 3.**
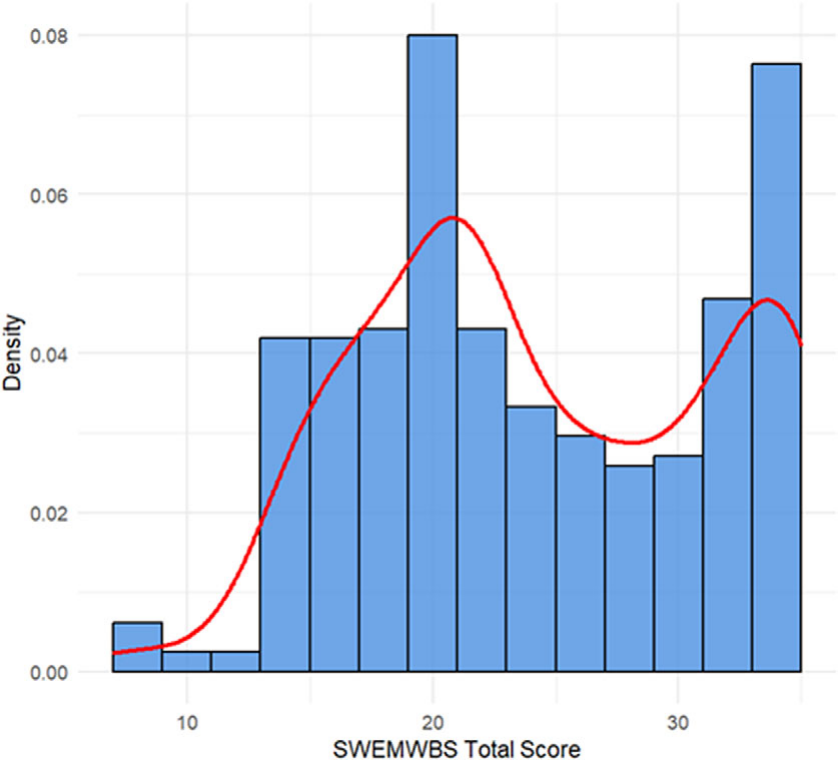
Histogram with density curve for SWEMWBS scores.

The overall mean SWEMWBS score was 24.39 (SD = 7.11), with observed values ranging from 7 to 35. Visual inspection of the distribution via *Q*–*Q* and histogram plot (Figures 2 and 3) indicated minor skewness (0.04) and moderate kurtosis (−1.09), suggesting slight bimodality and mild ceiling effects. These characteristics support the use of nonparametric and cluster-based methods to capture latent subgroups in mental well-being.

### Gender, education and sleep differences

Mental well-being scores varied significantly across gender and education levels. Males had significantly higher mean SWEMWBS scores (*M* = 25.66, SD = 7.12) than females (*M* = 22.99, SD = 6.84), with a *t*-test indicating a highly significant difference (*t*(404) = 3.84, *p* < .001) (Table 3).

**Table 3.** Comparison of mental well-being by gender and education, and sleep patterns by alcohol use (*N =* 406)

Variable/Group	*N*	Mean (SD)/*n* (%)	Test type	Test statistic	*p*-value
**Mental well-being (SWEMWBS)**					
***Gender***					
Male	213	25.66 (7.12)			
Female	193	22.99 (6.84)	*t*-test	*t* = 3.84	< .001
***Education of the head***	406		ANOVA	*F* = 10.55	< .001
Illiterate	46	19.41 (5.89)			
Primary school	125	22.66 (5.86)			
Middle school	80	24.98 (6.53)			
High school	91	26.42 (7.29)			
Intermediate/diploma	41	28.95 (6.63)			
Graduate	21	25.62 (9.30)			
***Sleep quality* vs. *alcohol consumption***			Chi-square	*χ* ^2^ = 2.073	.150
Alcohol users – good sleep	135	119 (88.1%)			
Non-users – good sleep	271	224 (82.7%)			
***Sleep duration* vs. *alcohol consumption***			Chi-square	*χ* ^2^ = 20.75	< .001
Alcohol users – ≥7 h sleep	135	75 (55.6%)			
Non-users – ≥7 h sleep	271	88 (32.5%)			

A one-way ANOVA revealed significant differences in well-being across levels of education of the household head (*F*(6, 399) = 10.55, *p* < .001), with scores increasing progressively from illiterate (*M* = 19.41) to intermediate/diploma holders (*M* = 28.95).

While sleep quality did not significantly differ between alcohol users and non-users (*χ* ^2^ = 2.073, *p* = .150), sleep duration showed a marked difference (*χ* ^2^ = 20.75, *p* < .001), with alcohol users more likely to report sleeping ≥7 h (55.6% vs. 32.5%).

### Correlation analysis

The Pearson correlation results (Table 4) showed that mental well-being (SWEMWBS score) was moderately and positively correlated with sleep duration (*r* = 0.46, *p* < .001) and education of the household head (*r* = 0.33, *p* < .001). In contrast, psychological distress indicators, stress (*r* = −0.38, *p* < .001), depression (*r* = −0.39, *p* < .001) and anxiety (*r* = −0.36, *p* < .001), showed significant negative correlations with mental well-being. These findings highlight the contribution of both behavioral (sleep and education) and emotional (stress, depression and anxiety) determinants to overall mental well-being.

**Table 4.** Pairwise Pearson correlations between key study variables (*r* and *p*-values)

Variable 1	Variable 2	*r*	*p-*value
SWEMWBS Score	Education level of the head	0.329	<.001
SWEMWBS Score	Sleep duration	0.462	<.001
SWEMWBS Score	Age	−0.053	.289
SWEMWBS Score	Monthly income score	0.069	.162
SWEMWBS Score	Stress	−0.382	<.001
SWEMWBS Score	Depression	−0.309	<.001
SWEMWBS Score	Anxiety	−0.369	<.001
Education level of the head	Sleep duration	0.293	<.001
Education level of the head	Age	−0.134	.007
Education level of the head	Monthly income score	0.040	.419
Education level of the head	Stress	−0.203	<.001
Education level of the head	Depression	−0.171	.001
Education level of the head	Anxiety	−0.184	< .001
Sleep duration	Age	−0.128	.010
Sleep duration	Monthly income score	0.025	.617
Sleep duration	Stress	−0.197	<.001
Sleep duration	Depression	−0.187	<.001
Sleep duration	Anxiety	−0.201	<.001
Age	Monthly income score	0.053	.291
Age	Stress	0.051	.309
Age	Depression	0.047	.348
Age	Anxiety	0.045	.367
Monthly income score	Stress	−0.148	.003
Monthly income score	Depression	−0.137	.006
Monthly income score	Anxiety	−0.127	.010
Stress	Depression	0.895	<.001
Stress	Anxiety	0.888	<.001
Depression	Anxiety	0.880	<.001

### Predictors of mental well-being: multiple regression analysis

**Figure 4.**
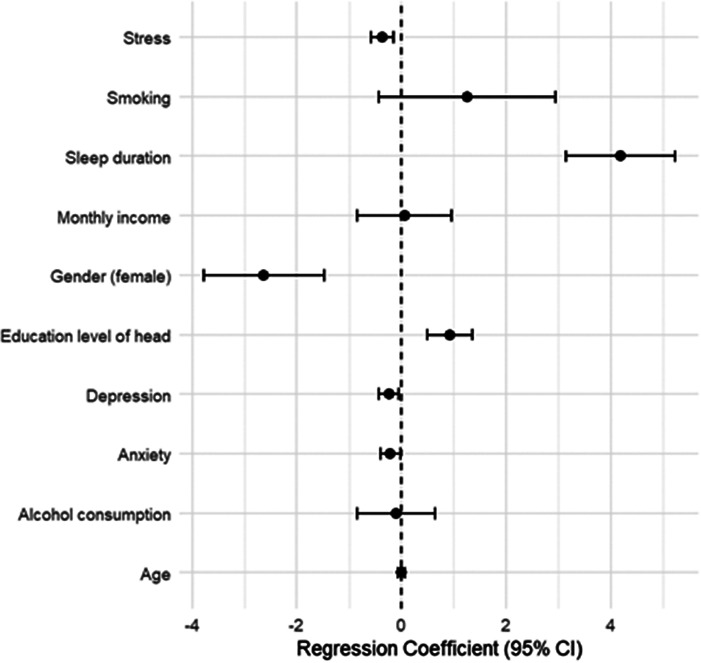
Forest plot of regression coefficients and 95% confidence intervals predicting mental well-being (SWEMB).

A multiple linear regression model (Table 5) was conducted to examine predictors of overall SWEMWBS scores. The model was statistically significant (*F*(10, 395) = 24.04, *p* < .001), explaining 37.8% of the variance in mental well-being (Adjusted *R* ^2^ = 0.363). Longer sleep duration was the positive predictor of well-being (*B* = 4.19, *p* < .001), followed by the education level of the household head (*B* = 0.92, *p* < .001). Lower stress scores were associated with higher well-being (*B* = −0.36, *p* = .001). Gender was also a significant predictor, with females scoring −2.63 points lower than males, independent of other variables (*B* = −2.63, <.001). Depression and anxiety demonstrated small but statistically significant negative associations with well-being. Other variables, including age, income, smoking and alcohol consumption, were not significant predictors in the adjusted model. Forest-plot visualization of these regression coefficients is presented in Figure 4.

**Table 5.** Multiple linear regression predicting mental well-being

Predictor variables	*B* (SE)	*p*-value
Gender (male vs. female)	−2.63 (0.59)	<.001***
Age (years)	0.001 (0.033)	.987
Monthly income score	0.06 (0.46)	.893
Education level of the head	0.92 (0.22)	<.001***
Present smoking	1.26 (0.86)	.143
Present alcohol consumption	−0.10 (0.38)	.791
Sleep duration (in hours)	4.19 (0.53)	<.001***
Stress score	−0.36 (0.11)	.001**
Depression score	0.24 (0.10)	.021*
Anxiety score	−0.21 (0.10)	.038*
Constant	15.16 (2.94)	<.001***

Significance codes: *p* < .05 (*), *p* < .01 (**), *p* < .001 (***).

### Cluster analysis of mental well-being profiles

**Figure 5.**
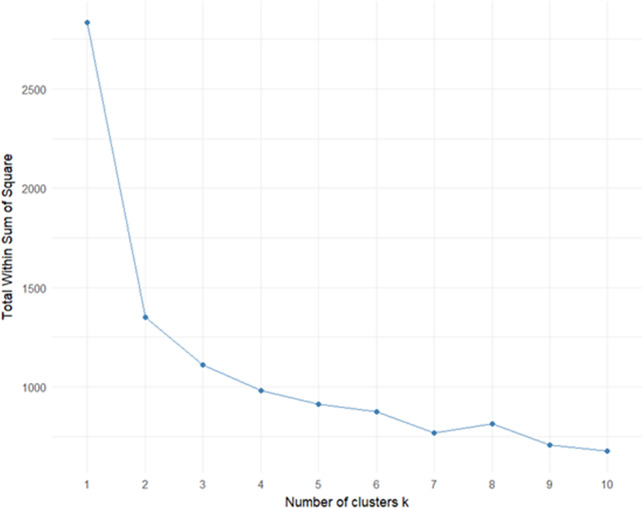
Optimal number of clusters determined using the Elbow method based on total within-cluster sum of squares (WSS). A noticeable inflection (elbow) is observed at *k = 3*, indicating that a three-cluster solution best balances model simplicity and explanatory power.

To uncover underlying typologies, *K*-means clustering was performed on the seven SWEMWBS item scores. The elbow method (Figure 5) indicated that a three-cluster solution was optimal, balancing parsimony and explanatory power. Both the silhouette coefficient and the Davies–Bouldin index confirmed that the three-cluster solution demonstrated superior cohesion and separation compared to other tested cluster solutions, supporting the selection identified by the elbow method.

Cluster-wise descriptive statistics for SWEMWBS total score, stress, depression, anxiety and each item are presented in Table 6.

**Table 6.** Cluster-wise descriptive statistics

Variable	Cluster 1	Cluster 2	Cluster 3
SWEMWBS (mean ± SD)	19.74 ± 3.30	20.21 ± 6.31	32.26 ± 2.74
Stress	3.39	16.43	0.58
Depression	1.86	16.32	0.73
Anxiety	3.20	16.88	0.72
Feeling optimistic	2.98	2.93	4.78
Feeling useful	2.93	2.87	4.81
Feeling relaxed	2.73	3.08	4.76
Dealing with problems	2.90	2.87	4.76
Thinking clearly	2.72	2.89	4.75
Feeling close to others	2.72	2.84	4.29
Making up my mind	2.77	2.73	4.10

The *K*-means clustering revealed three distinct subgroups based on participants’ SWEMWBS responses: Cluster 1 (High well-being): This group showed consistently high scores across all seven well-being indicators, suggesting strong emotional and psychological functioning. Cluster 2 (Moderate well-being): Participants in this cluster reported average to slightly below-average scores, indicating moderate mental well-being. Cluster 3 (Low well-being): This subgroup demonstrated reduced scores on several SWEMWBS items, pointing to lower levels of perceived mental well-being.

**Figure 6.**
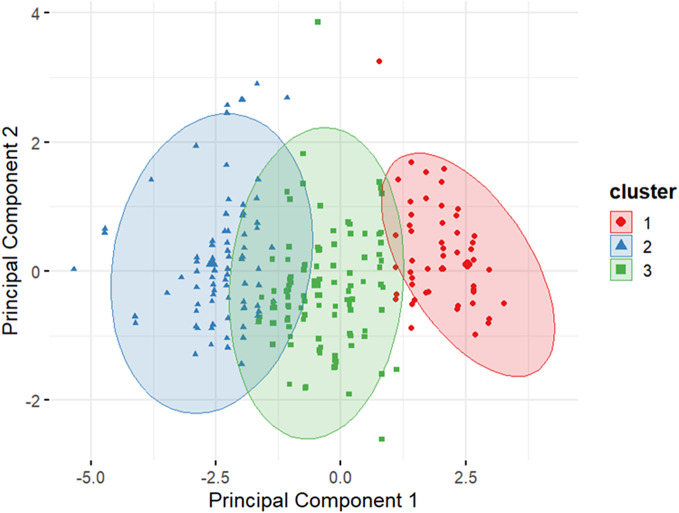
*K*-means-based mental well-being clusters (*k* = 3) derived from SWEMWBS item scores, visualized using principal component analysis. Each point represents a participant, colored by cluster. Ellipses represent 95% confidence areas for each cluster.

The cluster map (Figure 6) demonstrates clear separation between these groups in 2D space, with relatively low within-cluster variability. These findings suggest meaningful differentiation in well-being profiles within the sample population.

ANOVA and chi-square tests confirmed statistically significant differences in mental well-being scores and gender distribution across the identified clusters (Table 7).

**Table 7.** Statistical test results comparing clusters

Test	DF	*F*/Chi-square value	*p*-value
SWEMWBS	2, 403	482.1	<.001
Gender	2	29.30	<0.001

### Cluster-wise SWEMWBS item comparison

**Figure 7.**
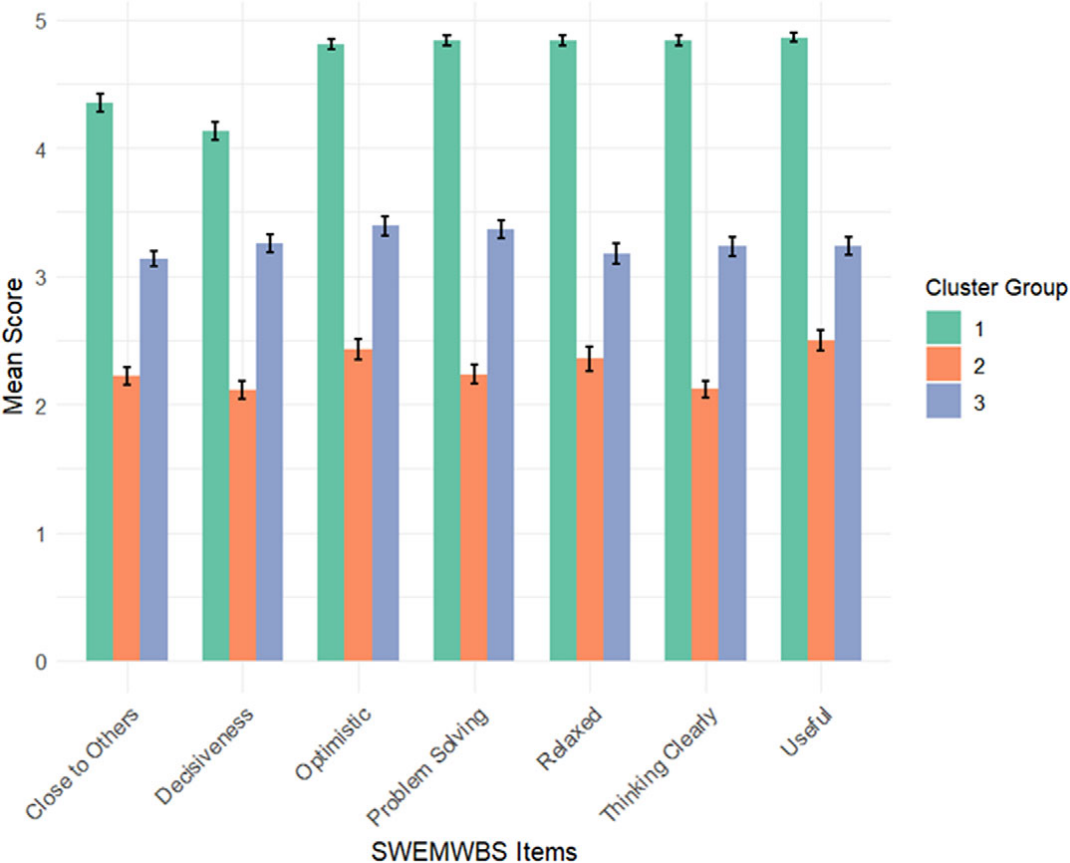
Cluster-wise comparison of mental well-being (SWEMWBS) item scores.

Figure 7 displays the mean scores of the seven items from the SWEMWBS across three identified mental well-being clusters derived through *k*-means clustering. Cluster 1 consistently shows the highest mean scores across all SWEMWBS items, suggesting individuals in this group have the strongest positive mental well-being profile. Notably, they score especially high on items such as “*Thinking Clearly*,” “*Feeling Relaxed*” and “*Feeling Optimistic about the Future.*” Cluster 2 exhibits the lowest mean scores across all items, indicating comparatively poorer mental well-being. Their scores are markedly low on “*Feeling Relaxed*,” “*Optimism*” and “*Thinking Clearly*,” pointing toward possible emotional distress or mental health challenges. Cluster 3 falls in between the other two groups, with moderate scores across most dimensions, representing a mixed or intermediate well-being profile. They show relatively balanced scores across items, without extreme highs or lows.

This cluster pattern reflects meaningful heterogeneity in mental well-being within the population. The differences across clusters may help in tailoring targeted mental health interventions and highlight the importance of identifying subgroups with varying needs.

## Discussion

This study explored mental well-being among adults residing in urban slums of Gorakhpur, India, using a cluster-based analytic approach applied to the SWEMWBS. Through *K*-means clustering, three distinct mental well-being profiles – High, Moderate and Low – were identified. These clusters reflect meaningful heterogeneity in the psychological functioning of urban slum residents, offering insights into patterns often overlooked in mean-based or regression-only analyses (Newson et al., 2020; Gao et al., 2023).

The three-cluster solution derived in this study is consistent with prior research applying similar techniques to different populations. For example, the UPRIGHT study in Europe used cluster analysis to categorize adolescents into subgroups based on resilience and well-being scores, identifying comparable “low-functioning” and “high-functioning” profiles (Las Hayas et al., 2019). Our findings expand this typology into an adult population within an LMIC urban slum context, where mental well-being segmentation is rarely applied despite substantial psychosocial burden (Maitra et al., 2015; Ezeh et al., 2017).

The cluster scores in our study suggest a polarized distribution, with the Low well-being group showing reduced scores across all SWEMWBS items, particularly on dimensions such as “feeling optimistic,” “thinking clearly” and “feeling relaxed.” These domains are often considered core to both hedonic and eudaimonic well-being (Tennant et al., 2007; Joshanloo, 2024), suggesting that individuals in this group experience difficulties not only with emotional states but also with cognitive clarity and purpose. In contrast, the High well-being group showed elevated functioning across all domains, suggesting psychological flourishing even in socioeconomically constrained environments (Yang et al., 2024). The Moderate group occupied a middle ground, with scores indicating vulnerability but not acute impairment (Ascher-Svanum et al., 2013).

This clear stratification offers strong empirical support for the dual-continua model of mental health, which posits that well-being and mental illness are distinct yet correlated dimensions (Slade, 2010). Indeed, individuals in the Low well-being group had significantly higher stress, depression and anxiety scores, but some in the Moderate group showed impaired well-being without severe symptoms (Shah et al., 2021; Pakpour et al., 2024). These findings align with earlier Indian studies, which revealed that substantial portions of low-income urban populations exhibit poor well-being in the absence of clinical psychiatric diagnoses (Maitra et al., 2015; Gautam et al., 2024). Our subgroup findings are also theoretically aligned with the dual-continua model, which predicts heterogeneous patterns of mental functioning even within populations sharing similar levels of socioeconomic adversity. Although all selected settlements shared broadly similar informal characteristics, variations in slum typology, such as tenure security, access to services or degree of municipal recognition, may influence mental well-being. Because slum-type information was not systematically recorded in this study, we were unable to analyze differences in well-being profiles across slum categories.

A notable and statistically robust finding was the gender-based difference in well-being profiles, with females disproportionately represented in the Low well-being group (*χ* ^2^ = 29.30, *p* < .001). This reinforces existing literature on the gendered burden of mental distress in Indian slums, where women often face layered vulnerabilities, including household responsibilities, limited autonomy and restricted access to health services (Abdulhadi et al., 2024; Koly et al., 2025). A 2021 study in Mumbai’s informal settlements similarly reported that women had significantly lower SWEMWBS scores than men, despite controlling for education and income (Kanougiya et al., 2024).

Education emerged as another strong and consistent predictor of well-being. The mental well-being score increased steadily with the household head’s education level, with the lowest mean observed among illiterate participants (*M* = 19.41) and the highest among those with intermediate or diploma-level education (*M* = 28.95). This mirrors findings from national data in England and from Australian cohorts, where higher educational attainment has been consistently linked with greater well-being, partly due to increased cognitive resources, health literacy and coping strategies (Carver et al., 2024; Gautam et al., 2024).

One of the most striking and policy-relevant findings from our regression analysis was the strong positive association between sleep duration and mental well-being (*B* = 4.19, *p* < .001). Participants who slept ≥7 h reported significantly better well-being scores, independent of gender, education or alcohol use (Ueno et al., 2025). Although the quality of sleep did not differ significantly between alcohol users and non-users, sleep duration did, with alcohol users more likely to report longer sleep. However, this finding should be interpreted cautiously, as longer sleep may sometimes reflect fatigue, low activity or other contextual factors in low-income populations (Park et al., 2015; Sirtoli et al., 2023).

The SWEMWBS was negatively correlated with psychological distress variables: stress, depression and anxiety. Stress emerged as the strongest negative predictor of well-being (*B* = −0.36, *p* = .001), followed by anxiety and depression. These results confirm earlier psychometric validations of the SWEMWBS, including in urban India and UK populations, where emotional distress accounted for a substantial proportion of variance in well-being scores (Shah et al., 2021; Pakpour et al., 2024). Our study extends these findings to a highly underrepresented population – urban slum dwellers – thereby enhancing the cross-cultural generalizability of these associations (Mahapatra et al., 2016).

Interestingly, income did not significantly predict well-being, which departs from conventional assumptions. This could be due to relative socioeconomic homogeneity in the sample (over 90% belonged to the lower and upper–lower Kuppuswamy categories), suggesting that income may only differentiate well-being across broader Socioeconomic status (SES) gradients but not within uniformly poor populations (Barry, 2009; Nolan, 2015).

These findings highlight that mental well-being varies considerably even within socioeconomically similar slum populations, consistent with prior evidence of substantial heterogeneity in psychological functioning among low-income urban groups (Maitra et al., 2015; Ezeh et al., 2017; Kanougiya et al., 2024). This calls for a more nuanced, data-driven segmentation approach in mental health programs. Rather than offering generalized services, policymakers and community health planners can develop cluster-specific interventions (Las Hayas et al., 2019; Gao et al., 2023). For example, the Low well-being group may benefit from intensive mental health screening, stress-reduction programs and female-focused outreach (Kirkbride et al., 2024). The Moderate group, which is often overlooked, might respond well to community-based resilience-building programs and psychoeducation (Slade, 2010). The High well-being group, though thriving, may still benefit from maintenance strategies to prevent decline, especially during crises (Holt-Lunstad, 2024).

Second, our study supports the integration of nonclinical, positive mental health measures like the SWEMWBS into routine public health surveillance in India. The tool’s brevity, strong reliability (*α* = 0.922) and cross-cultural adaptability make it suitable for large-scale, low-literacy populations. Combined with mobile data collection and cluster-based modeling, this approach can help identify at-risk subpopulations more efficiently (Blumberg and Luke, 2009; Billingham et al., 2013).

Lastly, our findings emphasize the growing need to incorporate digital and behavioral variables like mobile phone literacy or sleep behavior into mental health assessments in urban India. As the country’s health ecosystem becomes increasingly digitized, capturing these correlates may help bridge service gaps through digital mental health interventions (Sun et al., 2022; Arias López et al., 2023; Venkatesh et al., 2025).

## Limitations

Despite its strengths, this study has several limitations that warrant consideration. The cross-sectional design precludes any causal inferences, making it difficult to determine the temporal direction of associations between predictors and mental well-being. All data were self-reported, which may introduce recall bias or social desirability effects particularly in sensitive domains such as substance use, psychological distress or sleep behaviors. The study was restricted to slum communities within Gorakhpur urban agglomeration, limiting generalizability to other urban, rural or non-slum populations across India. Furthermore, the exclusive use of quantitative tools, while methodologically robust, may have overlooked important contextual and cultural dimensions of well-being that qualitative methods could capture. The scope of independent variables was also limited, omitting potentially relevant factors such as employment status, social support, nutrition, physical activity and neighborhood quality. In particular, important determinants of mental well-being, such as employment status, perceived social support, physical activity patterns and neighborhood safety, were not included in our models due to data constraints. These factors are known to influence psychological functioning in low-income urban settings, and their exclusion may have limited the explanatory power of our analyses. Additionally, several vulnerable groups, such as pregnant or lactating women, cognitively impaired individuals and those without smartphone access, were excluded, potentially underestimating mental health disparities in these subpopulations. The requirement that participants possess an Android phone, as part of the larger digital health project framework, may have unintentionally excluded some of the most socioeconomically marginalized residents of slum communities. Individuals without smartphones often have lower income, lower education and reduced access to health services, meaning that our findings may underrepresent those with the greatest vulnerability. This selection criterion therefore limits generalizability and may have resulted in conservative estimates of poor mental well-being. Methodologically, while *K*-means clustering offered useful subgroup identification, it assumes equal variance and cluster shapes, which may not fully reflect real-world complexity. Lastly, the observed bimodality and ceiling effects in SWEMWBS scores suggest that the scale, while psychometrically sound, may not capture subtle gradations in mental wellness, particularly at the higher end of the spectrum.

## Policy implications

Stratified mental health programming: Mental well-being is not uniform even within socioeconomically disadvantaged groups. Cluster-based profiling can guide tiered interventions targeting “low well-being” individuals with focused psychosocial support and preventive strategies for the “moderate” group. Gender-sensitive mental health services: The overrepresentation of women in the low well-being cluster highlights the need for women-centric community mental health programs, addressing gender-specific stressors, safety concerns and caregiving burdens. Education-linked interventions: As educational status strongly predicted well-being, mental health literacy may be integrated into adult education and skilling initiatives to strengthen coping capacity in slum communities. Sleep as a public health target: Given the strong link between sleep duration and mental well-being, urban health programs should incorporate sleep hygiene education into routine ASHA and Anganwadi outreach activities. Integration of digital literacy: the inclusion of mobile device proficiency suggests the potential to embed digital tools in slum-based mental health interventions. Expanding digital access and training can support outreach and self-care. Strengthening community-based platforms: Accredited Social Health Activist (ASHA) workers, Anganwadi workers and Urban Primary Health Centers (UPHCs) can integrate brief mental well-being screening (e.g., SWEMWBS) into routine visits, enabling early identification and referral for individuals in the low well-being cluster. Cluster-specific intervention packages: The low well-being group may benefit from stress management sessions, women-focused support groups and UPHC-based referral pathways. The moderate group may respond to resilience-building workshops delivered through Anganwadi or community centers. High well-being individuals could be engaged as peer champions. Embedding within existing policies: Cluster-based profiling can be incorporated into the National Urban Health Mission frameworks to guide prioritization, resource allocation and targeted mental health initiatives. Leveraging community collectives: Self-help groups, slum development committees and nongovernmental organizations can facilitate psychoeducation and promote supportive social environments. Addressing digital exclusion: Given uneven smartphone access, digital mental health programs should be paired with offline, community-based delivery channels to avoid widening inequalities.

## Conclusion

This study provides novel insights into the heterogeneity of mental well-being among adults living in urban slums of Gorakhpur, India, using a person-centered cluster analysis approach with the SWEMWBS. The identification of three distinct well-being profiles – High, Moderate and Low – demonstrates that psychological functioning varies substantially within socioeconomically similar populations. Key predictors, such as gender, education level, sleep duration and psychological distress, highlight the complex interplay of social and behavioral determinants in shaping mental wellness. These findings reinforce the importance of moving beyond average-based metrics to uncover latent subgroups that may benefit from tailored mental health interventions. Importantly, this study supports the integration of positive mental health measures and cluster-based segmentation into public mental health strategies for underserved communities. Addressing well-being disparities through context-specific, stratified approaches may significantly enhance the effectiveness and equity of mental health promotion efforts in rapidly urbanizing LMIC settings.

## Data Availability

The datasets generated and analyzed during the study are available from the corresponding author upon reasonable request.
