# Supplementary material for: Uncovering mental well-being profiles in urban slums of Gorakhpur, India: A cluster-based approach using SWEMWBS
Source: Glob Ment Health (Camb). 2026 Jan 26;13:e18. doi: 10.1017/gmh.2026.10132 (PMC12893872; doi:10.1017/gmh.2026.10132)
Supplement: Peer review history

# Reviewer report: Review: Uncovering mental well-being profiles in urban slums of Gorakhpur, India: A cluster-based approach using SWEMWBS — R1/PR2

**DOI:** 10.1017/gmh.2026.10132.pr2

**Authors:** Amjed Ali

**Affiliations:** Geography , Central University of Haryana, India

The manuscript is suitable for publication as submitted.

# Editor report: Recommendation: Uncovering mental well-being profiles in urban slums of Gorakhpur, India: A cluster-based approach using SWEMWBS — R1/PR3

**DOI:** 10.1017/gmh.2026.10132.pr3

**Affiliations:** Pravara Institute of Medical Sciences, India

Authors have addressed all the reviewer comments.

# Editor report: Decision: Uncovering mental well-being profiles in urban slums of Gorakhpur, India: A cluster-based approach using SWEMWBS — R1/PR4

**DOI:** 10.1017/gmh.2026.10132.pr4

**Affiliations:** London School of Hygiene & Tropical Medicine, United Kingdom of Great Britain and Northern Ireland

No accompanying comment.
